# Epidemiology and Management of Pediatric Group A Streptococcal Pneumonia With Parapneumonic Effusion: An Observational Study

**DOI:** 10.1097/INF.0000000000004418

**Published:** 2024-08-09

**Authors:** Emily A. Lees, Thomas C. Williams, Robin Marlow, Felicity Fitzgerald, Christine Jones, Hermione Lyall, Alasdair Bamford, Louisa Pollock, Andrew Smith, Theresa Lamagni, Alison Kent, Elizabeth Whittaker

**Affiliations:** * From the Department of Paediatrics, University of Oxford, Children’s Hospital Oxford, Oxford, United Kingdom; † Fitzwilliam College, University of Cambridge, Cambridge, United Kingdom; ‡ Department of Child Life and Health, University of Edinburgh, Edinburgh, United Kingdom; § Bristol Royal Hospital for Children, University Hospitals Bristol and Weston NHS Foundation Trust, Bristol, United Kingdom; ¶ Bristol Vaccine Centre, Schools of Population Health Sciences and of Cellular and Molecular Medicine, University of Bristol, Bristol, United Kingdom; ∥ Department of Paediatrics, Imperial College Healthcare NHS Trust, London, UK United Kingdom; ** Section of Paediatric Infectious Disease, Department of Infectious Disease, Imperial College London, London, United Kingdom; †† Faculty of Medicine and Institute for Life Sciences, University of Southampton, Southampton, United Kingdom; ‡‡ NIHR Southampton Clinical Research Facility and NIHR Southampton Biomedical Research Centre, University Hospital Southampton NHS Foundation Trust, Southampton, United Kingdom; §§ Department of Infectious Diseases, Great Ormond Street Hospital NHS Foundation Trust, London, United Kingdom; ¶¶ Infection, Immunity, and Inflammation Department, UCL Great Ormond Street Institute of Child Health, London; ‖‖ Department of Paediatric Infectious Diseases and Immunology, Royal Hospital for Children, Glasgow, United Kingdom; *** College of Medical, Veterinary and Life Sciences, Glasgow Dental School, University of Glasgow, Glasgow, United Kingdom; ††† Healthcare-Associated Infection & Antimicrobial Resistance Division, UK Health Security Agency, London, United Kingdom.; Children’s NHS Foundation Trust; Children’s NHS Foundation Trust; Barts Health NHS Trust; University Hospitals Bristol and Weston NHS Foundation Trust; University Hospitals Bristol and Weston NHS Foundation Trust; University Hospitals Bristol and Weston NHS Foundation Trust; University Hospitals Bristol and Weston NHS Foundation Trust; Cambridge University Hospitals NHS Foundation Trust; Cambridge University Hospitals NHS Foundation Trust; Guy’s and St Thomas’ NHS Foundation Trust; Newcastle Hospitals NHS Foundation Trust; Great Ormond Street Hospital for Children NHS Foundation Trust; Great Ormond Street Hospital for Children NHS Foundation Trust; Great Ormond Street Hospital for Children NHS Foundation Trust; Great Ormond Street Hospital for Children NHS Foundation Trust; Great Ormond Street Hospital for Children NHS Foundation Trust; Great Ormond Street Hospital for Children NHS Foundation Trust; Great Ormond Street Hospital for Children NHS Foundation Trust; Kings College Hospital NHS Foundation Trust; University Hospitals of Leicester NHS Trust; University Hospitals of Leicester NHS Trust; Leeds Teaching Hospitals NHS Trust; Manchester University NHS Foundation Trust; Manchester University NHS Foundation Trust; Manchester University NHS Foundation Trust; Norwich and Norfolk University Hospitals NHS Foundation Trust; Norwich and Norfolk University Hospitals NHS Foundation Trust; Nottingham University Hospitals NHS Trust; University Hospitals Sussex NHS Foundation Trust; University Hospitals Sussex NHS Foundation Trust; Guy’s and St Thomas’ NHS Foundation Trust; Belfast Health and Social Care Trust; Belfast Health and Social Care Trust; Belfast Health and Social Care Trust; NHS Greater Glasgow and Clyde; Sheffield Children’s NHS Foundation Trust; St George’s University Hospitals NHS Foundation Trust; St George’s University Hospitals NHS Foundation Trust; St George’s University Hospitals NHS Foundation Trust; Imperial College Healthcare NHS Trust; Imperial College Healthcare NHS Trust; Imperial College Healthcare NHS Trust; Imperial College Healthcare NHS Trust; Imperial College Healthcare NHS Trust; University Hospital Southampton NHS Foundation Trust; University Hospital Southampton NHS Foundation Trust; University Hospital Southampton NHS Foundation Trust; University Hospital Southampton NHS Foundation Trust; University Hospital Southampton NHS Foundation Trust; University Hospital Southampton NHS Foundation Trust; Oxford University Hospitals NHS Foundation Trust; Oxford University Hospitals NHS Foundation Trust; Oxford University Hospitals NHS Foundation Trust; Public Health Scotland; Public Health Scotland; UK Health Security Agency; UK Health Security Agency

**Keywords:** Group A streptococcus, empyema, parapneumonic effusion, vaccination, antimicrobial stewardship

## Abstract

**Background:**

During autumn/winter 2022, UK pediatricians reported an unseasonal increase in invasive group A streptococcal infections; a striking proportion presenting with pneumonia with parapneumonic effusion.

**Methods:**

Clinicians across the United Kingdom were requested to submit pseudonymized clinical data using a standardized report form for children (<16 years) admitted between September 30, 2022 and February 17, 2023, with microbiologically confirmed group A streptococcal pneumonia with parapneumonic effusion.

**Results:**

From 185 cases submitted, the median patient age was 4.4 years, and 163 (88.1%) were previously healthy. Respiratory viral coinfection was detected on admission for 101/153 (66.0%) children using extended respiratory pathogen polymerase chain reaction panel. Molecular testing was the primary method of detecting group A streptococcus on pleural fluid (86/171; 50.3% samples). Primary surgical management was undertaken in 171 (92.4%) children; 153/171 (89.4%) had pleural drain inserted (96 with fibrinolytic agent), 14/171 (8.2%) had video-assisted thoracoscopic surgery. Fever duration after admission was prolonged (median, 12 days; interquartile range, 9–16). Intravenous antibiotic courses varied in length (median, 14 days; interquartile range, 12–21), with many children receiving multiple broad-spectrum antibiotics, although evidence for additional bacterial infection was limited.

**Conclusions:**

Most cases occurred with viral coinfection, a previously well-recognized risk with influenza and varicella zoster, highlighting the need to ensure routine vaccination coverage and progress on vaccines for other common viruses (eg, respiratory syncytial virus, human metapneumovirus) and for group A streptococcus. Molecular testing is valuable to detect viral coinfection and confirm invasive group A streptococcal diagnosis, expediting the incorporation of cases into national reporting systems. Range and duration of intravenous antibiotics administered demonstrated the need for research on the optimal duration of antimicrobials and improved stewardship.

*Streptococcus pyogenes* (group A streptococcus, or GAS) is responsible for a broad spectrum of clinical diseases. This includes superficial, invasive group A streptococcal (iGAS) and postinfectious disease syndromes and is an important cause of morbidity and mortality in children globally.^1,2^ There are over 200 ‘*emm*’ strains of GAS identified to date. In the United Kingdom, a sublineage of *emm1* (M1_UK_) with increased toxigenicity was identified in 2015. This is currently the dominant circulating strain in the United Kingdom and is associated with pharyngitis, scarlet fever and iGAS infection; demonstrating intense transmission in a school environment.^3^ Global spread of M1_UK_ is occurring with cases reported in Australia, United States, Canada and the Netherlands.^4^

GAS infections in the United Kingdom usually peak in spring; however, during the 2022–2023 season, multiple countries worldwide described out-of-season peaks of scarlet fever and iGAS.^5–7^ In the United Kingdom, these cases are notifiable to local public health authorities. Between September 12, 2022 and February 17, 2023, there were increased notifications for iGAS (522 cases) in children under 18 years in England, compared to the whole of the last “peak” season September 2017 to August 2018 (205 cases),^8^ and in Scotland, 60 pediatric iGAS were reported. This comes after a period of decreased GAS activity during the first 2 years of the severe acute respiratory syndrome coronavirus 2 pandemic.^5^ In the pediatric population in England, the highest rates of iGAS between September 2022 and February 2023 were in 1- to 4-year olds (9.8 cases/100,000 population), followed by 7.8 and 4.8 cases/100,000 population in <1 year and 5- to 9-year olds, respectively. These are marked increases in yearly prepandemic rates of 0.5, 1.1 and 0.3 cases/100,000 population.^8,9^ It has been proposed that this demonstrates a differing demographic at risk; children who are older at their first GAS exposure, with reduced incremental immunity due to exposure for these children, with enhanced hygiene measures during the severe acute respiratory syndrome coronavirus 2 pandemic.^5^

An increased incidence of iGAS in the form of pneumonia with effusion or empyema was noted by UK pediatricians in autumn 2022.^10^ The descriptors “parapneumonic effusion” and “empyema” represent a continuum from clear pleural fluid with few white blood cells, to septations and organized collection of pus complicating bacterial pneumonia.^11^ There are no data correlating the stages along this continuum with management strategy; therefore, we will refer to “parapneumonic pleural effusion” (PPE) to include all of these stages.

Data on pediatric PPE in the post-pneumococcal vaccine era suggest that *Streptococcus pneumoniae* remains a predominant causative agent,^12^ alongside other pathogens such as *Staphylococcus aureus* and GAS. Those with GAS PPE have an increased likelihood of Pediatric Intensive Care Unit (PICU) admission versus pneumococcal PPE.^13,14^ There is no agreed standard treatment for childhood PPE, with no clear difference in outcomes between chest drain with fibrinolysis and video-assisted thoracoscopic surgery (VATS), and treatment is often dependent on local availability and clinician preference.^15^ For antibiotic management, a lack of prospective studies means no uniform UK pediatric guidance exists on choice of agent, narrowing of spectrum, route of administration and duration of treatment for GAS empyema.^11^

Here, we describe the presentation, investigations, management and outcomes of the largest single cohort of children with GAS pneumonia with parapneumonic effusion.

## METHODS

This is a retrospective case series of children admitted to UK hospitals between September 30, 2022 and February 17, 2023, with pseudonymized data collected from medical records in each center. Clinicians were invited to submit cases using a secure online form via national subspeciality networks (British Paediatric Allergy, Immunology and Infection Group, British Paediatric Respiratory Society and Paediatric Critical Care Society). The project was registered locally as a multi-site service improvement project to inform guideline development and management of cases.

### Case Definition

Children (<16 years old) admitted to any NHS hospital with confirmed GAS pneumonia with parapneumonic pleural effusion (PPE) as defined by meeting criteria (A) and (B):

- PPE diagnosis: radiological confirmation of an effusion or collection
- iGAS infection: microbiologic culture or molecular confirmation [specific polymerase chain reaction (PCR) or broad range 16S rDNA PCR] of GAS on blood or pleural fluid

### Statistical Analysis

Continuous variables are presented as median, range and interquartile range. Categorical variables are presented as percentages and compared using χ^2^ test. Statistical analysis was done using GraphPad Prism version 9.5.0, with a 2-sided *P* value of <0.05 considered statistically significant. Methods for generation of figures on antibiotic use and viral coinfection are outlined in Supplemental Digital Content 2 and 3, http://links.lww.com/INF/F580.

## RESULTS

Data were submitted for 185 children admitted to 22 UK hospitals with confirmed GAS pneumonia with PPE between September 30, 2022 and February 17, 2023. Cases were reported from across the United Kingdom, with 163 in England, 18 in Scotland and 4 in Northern Ireland. Case numbers submitted per hospital ranged from 1 to 22 (median 7).

### Clinical Characteristics of Patients

Median age was 4.4 years (IQR, 2.5–6.3), 84 (45.4%) were female and 138 (71.1%) were of White ethnicity (Table 1). Most patients were previously healthy (163/185, 88.1%). Children most frequently presented with fever (95.1%), cough (74.6%), and difficulty breathing (65.9%). Preceding admission, 84 (45.4%) children had received prior medical review during their illness (either via General Practitioner or the emergency department), with 17/84 children (20.2%) having 2 visits and 4 children (2.2%) 3 visits. Oral antibiotics appropriate for GAS infection were received by 31 (16.8%) children before admission. The commonest diagnosis was unilateral pneumonia with PPE (165, 89.2%), with 35 (18.9%) children having associated streptococcal toxic shock syndrome (STSS).

**TABLE 1.** Demographics and Presenting Features (Total Cohort = 185)

	Denominator	n (%)/Median	n (%)/Range (IQR)
Female	185	84 (45.4)	
Age (years)	185	4.4 y	0.2–15.8 (2.5–6.3)
Age breakdown (years)	185		
<1		11 (5.9)	
1–4		89 (48.1)	
5–9		71 (38.4)	
10–14		13 (7.0)	
≥15		1 (0.5)	
Ethnicity	185		
White		138 (71.1)	
Asian or Asian British		26 (14.1)	
Black, African, Caribbean or Black British		8 (4.3)	
Mixed		1 (0.5)	
Other		12 (6.5)	
Comorbidities (total)	185	22 (11.9)	
Respiratory		6 (3.2)	
Neurological/developmental		6 (3.2)	
Cardiac		3 (1.6)	
Prematurity (<37 weeks’ gestation)		3 (1.6)	
Other		4 (2.2)	
Fully immunized to UK schedule*†	185		
Yes		138 (74.6)	
No		5 (2.7)	
Unknown		42 (22.7)	
Prior medical review during this illness (GP/ED)	185	84 (45.4)	
Prior oral antibiotics	185	31 (16.8)	
Symptoms	185	Presenting	During admission
Fever		176 (95.1)	180 (97.3)
Cough		138 (74.6)	152 (82.2)
Difficulty in breathing		122 (65.9)	141 (76.2)
Lethargy		80 (43.2)	97 (52.4)
Coryza		59 (31.9)	67 (36.2)
Rash		57 (30.8)	82 (44.3)
Vomiting		54 (29.2)	62 (33.5)
Sore throat		32 (17.3)	38 (20.5)
Diarrhea		22 (11.9)	29 (15.7)
Muscle aches		12 (6.5)	14 (7.6)
Headache		4 (2.2)	5 (2.7)
Chest pain		1 (0.5)	1 (0.5)
Seizure		0 (0)	2 (1.1)
Admitting diagnoses	185		
Unilateral pneumonia with parapneumonic effusion		165 (89.1)	
- Left-sided	165	80 (48.5)	
- Right sided	165	85 (51.5)	
Bilateral pneumonia with parapneumonic effusion		20 (10.8)	
Toxic shock syndrome		35 (18.9)	
Group A streptococcal bacteremia		50 (27.0)	

* UK Health Security Agency. Chapter 11: The UK Immunisation schedule. In: *The Green Book* 2022.

† Excluding influenza vaccination https://www.gov.uk/government/publications/immunisation-schedule-the-green-book-chapter-11.

GP/ED indicates general practitioner/emergency department.

### Clinical Course, Interventions, and Outcome

Admissions had a median duration of 16 days (IQR, 12–20; Table 2). PICU admission was required for 116 (64.3%) children, with median length of stay of 5 days (IQR, 3–9 days). Eighty-two (44.3%) children required intubation and ventilation, and 66 (35.7%) required vasoactive medications. Duration of fever after admission was prolonged, at a median of 12 days (IQR, 9–16 days).

**TABLE 2.** Clinical Management and Interventions

	Denominator	n (%)/median	Range (IQR)
Length of admission (days)	180*	16	2-66 (12–20)
PICU admission	185	119 (64.3)	
PICU admission duration (days)	116†	5	1–39 (3–9)
Ventilatory support	185		
None		30 (16.2)	
Low flow oxygen		35 (18.9)	
High flow oxygen		38 (20.5)	
Intubated and ventilated		82 (44.3)	
Vasoactive support required	185	66 (35.7)	
Ultrasound completed	185	158 (85.4)	
Simple effusion	158	79 (50.0)	
Loculated effusion		79 (50.0)	
CT performed during admission	185	50 (27.0)	
Primary surgical intervention	185		
Pleural tap		1 (0.5)	
Pleural drain		57 (30.8)	
Pleural drain + fibrinolytics		96 (51.9)	
VATS		14 (7.6)	
VATS + fibrinolytics		2 (1.1)	
Open thoracotomy		1 (0.5)	
None (antibiotic treatment only)		11 (5.9)	
Died prior to procedure		3 (1.6)	
Duration drain in situ (days)	152‡	7	1–22 (5–9)
Number of doses of fibrinolytic	98	6	1–28 (5–6)
Secondary intervention	171	28 (16.4)	
Pleural drain	28	12 (42.9)	
Pleural drain + fibrinolytics		1 (3.6)	
VATS		13 (46.4)	
Open thoracotomy		2 (7.1)	
Complications (if received intervention)	171	32 (18.7)	
Pneumothorax	32	21 (65.6)	
Hydropneumothorax		3 (9.4)	
Bronchopulmonary fistula		4 (12.5)	
Surgical emphysema		2 (6.3)	
Other		2 (6.3)	
Total fever duration (days)	158§	16	3–73 (12–19)
Fever duration post–admission (days)	158§	12	0–60 (9–16)
IV antibiotic duration (days)	176	14	1–71 (12–21)
Oral antibiotic duration (days)	170	14	0–42 (14–28)
Total antibiotic duration (days)‖	170	32	14–79 (26–42)
IV immunoglobulin received¶	185	31 (16.8)	
Outcome	185		
Discharged on IV or oral antibiotics		158 (85.4)	
Repatriated to local hospital		18 (9.7)	
Remains inpatient at tertiary center		1 (0.5)	
Died		8 (4.3)	

* Excluding 4 children who died within 24 hours of admission and one child remaining inpatient.

† Excluding one child who died within 24 hours admission and 2 with incomplete data.

‡ Excluding one child who died within 24 hours of admission.

§ Excluding seven children who died prior to defervescence and 20 with incomplete data.

‖ See Supplementary methods for calculation of antibiotic duration.

¶ NHS England Immunoglobulin Expert Working Group. Commissioning Criteria Policy for the use of therapeutic immunoglobulin (Ig) England, 2021. https://www.england.nhs.uk/wp-content/uploads/2021/12/cpag-policy-for-therapeutic-immunoglobulin-2021-update.pdf.

Eleven (5.9%) children recovered following intravenous (IV) antibiotic therapy without further intervention. A pleural drain was inserted in 153/185 (82.7%) children with fibrinolytics given in 96/153 (62.7%), VATS was the primary intervention in 16/185 (8.6%) children (see Consort chart, Supplemental Digital Content 4, http://links.lww.com/INF/F580). Surgical intervention occurred within 48 hours of admission in 130/171 (76.0%) children. A lower proportion (nonsignificant) of children required a second intervention if pleural drain with fibrinolytics versus pleural drain alone was the primary intervention [14/96 (14.6%) versus 14/57 (24.6%)]. No children who underwent VATS as their first procedure required a second intervention. Complications were common, occurring in 32/171 (18.7%) children who had received surgical intervention; this was most frequently pneumothorax (21/32, 65.6%).

**FIGURE 1.**
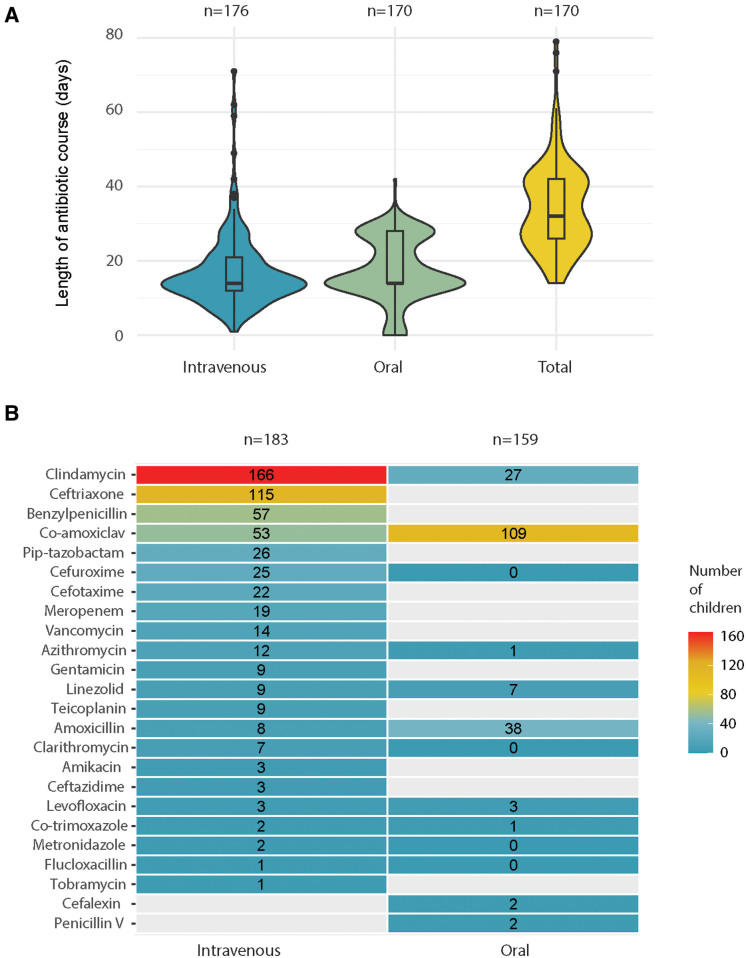
Antibiotic administration in the study cohort. A: Length of antibiotic course. Length of intravenous, oral, and total antibiotic course for children 0–16 years old in the cohort, showing median and interquartile range for each category. Participants with incomplete information on the length of course omitted from each respective violin plot. B: Antimicrobial agents administered. Heatmap showing number of participants who received each of the antimicrobial agents shown. Agents without an intravenous or oral route of administration shaded out in gray.

The majority (118/170; 69.4%) of children received >28 days of antibiotic treatment (Fig. 1A), with a median duration of IV treatment for 14 days (IQR, 12–21), followed by a median of 14 days oral therapy (IQR, 14–28). A wide range of antibiotics were used (Fig. 1B), with some children receiving up to 7 different intravenous antibiotics during their illness. During admission, probable bacterial secondary/coinfection was identified in 8 (4.3%) children, including 3 presumed coagulase-negative staphylococcal central venous catheter infections, 3 ventilator-associated pneumonias and 2 Gram-negative bacteremias. IV immunoglobulin was received by 31/35 children reported to have STSS, 3 of whom died.

Three months after the study close, 158 (85.4%) children had been discharged (139 on oral antibiotics, 18 on ambulatory IV antibiotics and 1 without further treatment), 18 (9.7%) had been repatriated to their local hospital due to clinical improvement (total length of stay not available for these cases) and 1 (0.5%) remained inpatient (under plastic surgery team following lower limb amputation for ischemia). Eight (4.3%) children died following admission, 6 of whom were bacteremic, with 5 deaths occurring within 48 hours of admission.

### Microbiologic Investigations and Viral Coinfection

Fifty of 185 (27.0%) children were bacteremic with GAS at presentation, and 73/171 (42.7%) had positive pleural cultures for GAS (24 of whom were also bacteremic; Table 3). The predominant method of microbiological diagnosis for pleural fluid samples was PCR; positive in 86/171 (50.3%) children sampled. Of pleural fluid cultures taken within 48 hours of admission, 69/121 (57.0%) were positive, versus 4/36 (11.1%) samples taken after 48 hours; *P*<0.0001. Twenty-eight of 76 (36.8%) children tested had a positive throat swab.

**TABLE 3.** Microbiological, Virological and Biochemical Parameters

	Denominator	n (%)/Median	Range (IQR)
GAS bacteremia	185	50 (27.0)	
Detection of GAS in pleural fluid	171	157 (91.8)	
Culture	157	71 (45.2)	
PCR		84 (53.5)	
Culture and PCR		2 (1.3)	
Throat swab available	185	76 (41.1)	
Culture positive for GAS	76	28 (36.8)	
GAS *emm* type available*	99	41 (41.4)	
1.0	41	34 (82.9)	
1.3		1 (2.4)	
12.0		4 (9.8)	
12.37		2 (4.9)	
ASOT result available	185	36 (19.5)	
<200	36	24 (66.7)	
200–400		5 (13.9)	
>400		7 (19.4)	
Extended respiratory viral PCR testing completed	185	153 (82.7)	
Children with respiratory virus identified on PCR	153	101 (66.0)	
Children with respiratory virus identified on other POCT	32	3 (9.4)	
Preceding VZV infection	185	5 (2.7)	
Number of codetected viruses per child having respiratory viral PCR	153		
0		52 (34.0)	
1		73 (47.7)	
2		19 (12.4)	
3		9 (5.9)	
Virus occurrences (total—including PCR/POCT/VZV):	146		
Adenovirus		16	
HMPV		36	
Influenza A		14	
Influenza B		1	
Parainfluenza		5	
Rhino/enterovirus		30	
RSV		32	
SARS-CoV-2		2	
Other coronavirus		5	
VZV		5	
Bacterial coinfection/secondary infection	185	8 (4.3)	
Presumed coagulase-negative staphylococci CVC infection		3 (1.6)	
Ventilator-associated pneumonia†		3 (1.6)	
Gram-negative organism bacteraemia‡		2 (1.1)	
Highest CRP (mg/L)	183§	292	39–652 (215–360)
Highest platelet count (×10*9 cells/L)	183§	731	72–2086 (503–915)

Extended respiratory viral PCR = panel testing for multiple viral pathogens (eg, Biomerieux Respiratory 2.1 panel), POCT = point of care test (RSV, influenza A/B, SARS-CoV-2), preceding VZV infection on clinician/parent report/blood PCR.

* From blood or pleural culture.

† *Enterobacter cloacae*, *Klebsiella pneumoniae*, additional organism not defined.

‡ *Pseudomonas aeruginosa*, ESBL-producing organism (not defined).

§ Excluding 2 children with missing data.

ASOT indicates antistreptolysin O titer; CRP, C-reactive protein; CVC, central venous catheter; ESBL, extended spectrum beta-lactamase.

*Emm* type was available for 41 isolates, with a predominance of *emm1* (35/41, 85.4%) followed by *emm*12 (6/41, 14.6%).

**FIGURE 2.**
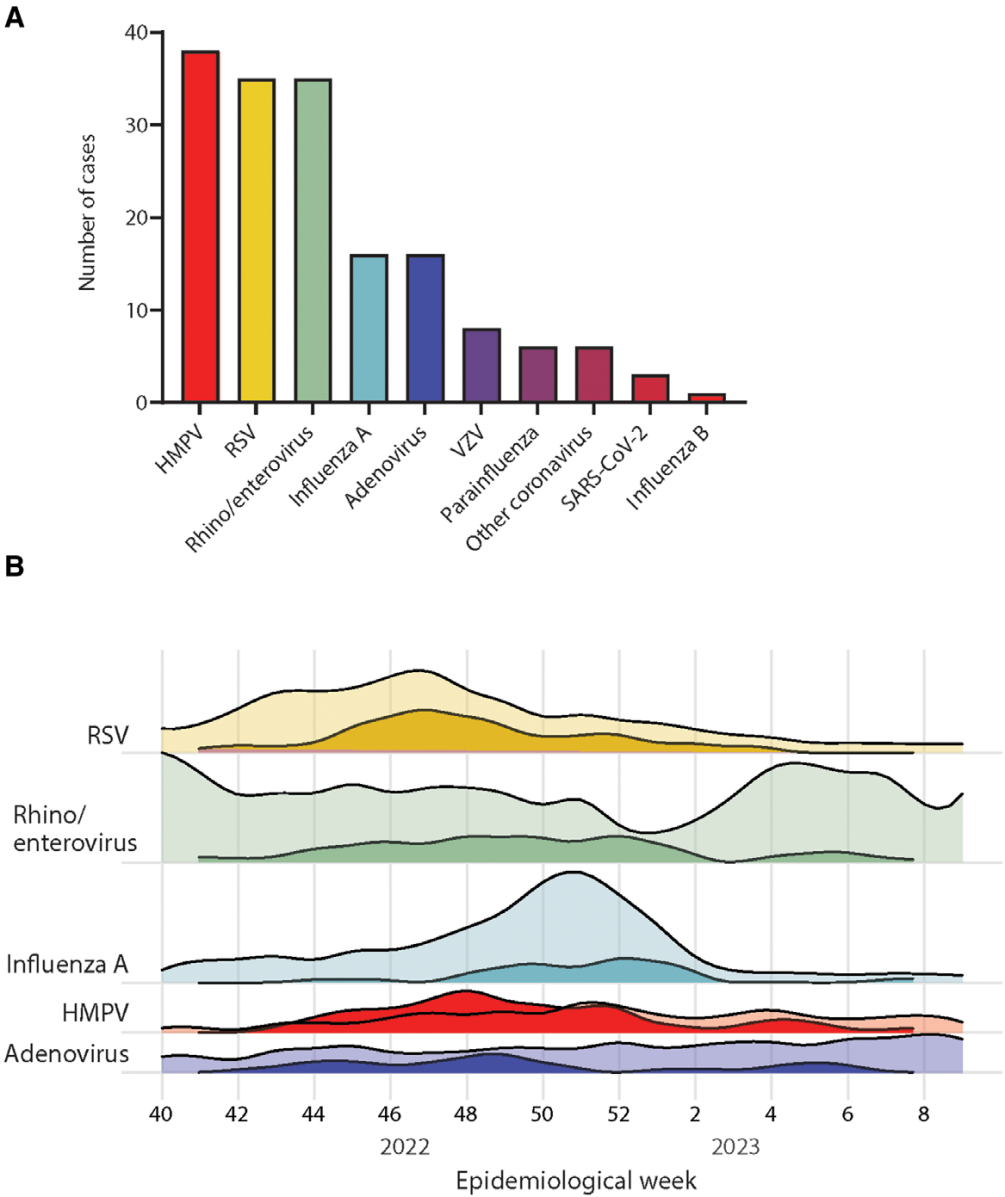
Viral codetections in the study cohort. A: Absolute counts of viruses detected in the cohort. B: Representation of the five most common viruses co-detected over time in the cohort (darker shade), plotted alongside semi-quantitative measure of UK virus incidence (lighter shade), calculated from UKHSA hospital surveillance data. In general, incidence of co-detected viruses in PPE cohort follows national incidence trends. Some viruses (eg, HMPV) appear over-represented relative to concurrent national incidence. Supplemental Digital Content 2 (Methods) contains detail on how relative incidence curves were calculated. SARS-CoV-2 indicates severe acute respiratory syndrome coronavirus 2.

Viral coinfection was present in many children, with human metapneumovirus (hMPV) the most frequent pathogen; detected in 36/185 (19.5%) children, followed by respiratory syncytial virus (RSV) and rhino/enterovirus identified in 32 (17.3%) and 30 (16.2%) children, respectively (Fig. 2A). An extended respiratory pathogen PCR panel was not sent for 32/185 (19.4%) children. Preceding varicella zoster virus (VZV) infection occurred in 5/185 (2.7%) children. Detected viruses reflected circulating respiratory viruses as per UK Health Security Agency (UKHSA) data (Fig. 2B), but an earlier/larger peak in hMPV was noted than was seen circulating more widely.

## DISCUSSION

We present a uniquely large case series of children with GAS pneumonia with PPE occurring over a short period of unseasonal high activity. The information gathered can be used to inform research and guideline development for future management of similar cases, with particular focus on opportunities for improved antimicrobial stewardship and assuring routine vaccination coverage, given the high rate of viral coinfections.

Age range of this cohort is consistent with that of cases reported to UKHSA for the 2022 to 2023 season, with relatively few cases occurring in infants <1 year of age. The majority (48.1%) of cases occurred in the 1–4 year age group, but a larger proportion of cases than expected (38.4%) were in the 5–9 year age group; an older demographic than previously described in iGAS PPE cases.^14^ Compared to the age distribution of iGAS bacteremia from any source, the <1 year age group is underrepresented in the pneumonia with PPE cohort.^16^ Patient ethnicity was consistent with UK population census data.^17^

These cases, almost two-thirds of whom required a stay on PICU, occurred in a predominantly healthy population, with only an 11.9% rate of comorbidities (predominantly respiratory or neurological conditions). This is far below the usual rate of comorbidities for children requiring PICU admission (46% in the United Kingdom),^18^ although higher than in a smaller all-cause PPE series (5.7%).^13^ Although 15 (8.1%) children were admitted to PICU for chest drain insertion without other organ dysfunction, the high proportion of children requiring mechanical ventilation (44.3%) and vasoactive medications (35.7%) indicates a population of critically ill patients. A high requirement for vasoactive support, consistent with a previous study^14^ indicates that those caring for children with GAS pneumonia need to be aware of the potential for sudden severe deterioration and need for escalation of care. The proportion of deaths in this study (8 children, 4.3% of cases, 4 of whom were under 5 years old) was low compared with a UKHSA case series of 147 children with iGAS LRTI (25%).^19^ This is in stark contrast to data on pneumococcal pneumonia with PPE, with reported case fatality rates of up to 5%.^20–22^ The majority of children in our cohort who died were bacteremic and died within 48 hours of admission, fitting the rapid deterioration and death described for iGAS in the community, and in the UKHSA study where 57% deaths occurred before hospital admission.^19^

There was no appreciable constellation of presenting symptoms which would reliably differentiate these cases of iGAS clinically from those of a viral infection; with fever, cough, difficulty in breathing, lethargy and rash being the predominant presenting features. This additionally appeared to be the case in the review of UK iGAS deaths, with some children experiencing a biphasic illness, or a rapid deterioration after a diagnosis with a presumed viral illness.^23^ Of note, although 66.0% of those tested had a positive respiratory viral PCR, only 31.9% were coryzal, suggesting that PCR data reflects either preceding or current respiratory viral infection. Rash and lethargy were the 2 most frequent symptoms to evolve over time; however, the cause of rash in these cases could be multifactorial, whether related to GAS exotoxin production, viral coinfection, or medication-related.

Most children had a pleural drain inserted, with or without fibrinolytics as their primary therapy; VATS as a primary treatment was center-specific, occurring in 4 centers as a primary intervention and 4 further centers as a secondary intervention. There was a surprisingly high rate of adverse events after surgical intervention (18.7%), versus 10% in a systematic review.^24^ These were largely pneumothorax, bronchopulmonary fistula or hydropneumothorax. It is not clear how many of these were related to intervention, versus the underlying lung/pleural pathology.^25^

As many pleural fluid diagnoses were made on PCR, *emm* types were available in a relatively low proportion of children (22.1%). In this study, 12/25 throat swabs taken from 84 children with molecular iGAS diagnosis from pleural fluid PCR were culture-positive. There is therefore potential value to obtaining noninvasive isolates in children with PPE to facilitate case finding and provide typing information, as requested as part of UKHSA’s incident investigation during this period of increased iGAS incidence.^26^

Of *emm* types identified, the largest group was *emm1*, which fits with the potential to cause invasive disease previously described with this strain;^1,4^ with sequencing of 2022 isolates from London, United Kingdom demonstrating association of *emm1* (predominantly M1_UK_) and the presence of superantigen genes *spea* and *spej* with invasive disease.^27^ The number of pleural culture-positive isolates was higher than in a previous UK study (46.5% versus 17%),^28^ but molecular methods increased pathogen recognition, as previously demonstrated in up to 75% culture-negative cases;^29^ advantageous in a climate of early antibiotic use in sepsis management. Not all centers have in-house molecular testing, therefore delays in obtaining a definitive molecular diagnosis may have contributed to the use of broader spectrum agents. Repurposing of GAS rapid antigen detection tests (usually used for pharyngeal samples) for pleural fluid showed promise as an alternative method of obtaining a rapid diagnosis in one study.^30^ As well as being important epidemiologically, pathogen identification is key to antimicrobial stewardship, to minimize toxicities and the risk of developing antimicrobial resistance with broader agents.^31^

Duration of fever post-admission was prolonged (median, 12 days), as previously reported for both GAS and *S. pneumoniae* PPE,^14^ with report in a large cohort of comparatively longer fever and hospital stay for GAS pneumonia,^32^ postulated to be due to extensive pleural inflammation.^14^ It is possible that continued fever contributed to unnecessary antibiotic escalation in a number of cases, with only 4.3% of children having confirmed bacterial secondary/coinfection; yet, many children receiving multiple antibiotics covering a much broader range of pathogens. The detection of a second bacterial pathogen was lower than had been seen in a retrospective all-cause PPE study of 1447 patients (9.4%).^33^ GAS is universally penicillin-sensitive, so children with proven GAS infection would be good candidates for de-escalation to benzylpenicillin (with additional clindamycin to inhibit toxin production in the acute stages); however, in this cohort, children largely remained on a third-generation cephalosporin alongside clindamycin. The advantage of ceftriaxone is its once daily administration for outpatient parenteral antibiotic therapy, or to reduce nursing workload. Previous guidance on length of IV therapy required is unclear and depends on adequate effusion drainage and the child’s clinical response.^11^ Minimum suggested total treatment course is 2 weeks (with at least 1 week IV)^34,35^ or up to 6 weeks in complicated infection. Early switch to oral antibiotics may be appropriate, with treatment failure rates comparable, or in one study higher for those on outpatient parenteral antibiotic therapy than oral antibiotics, due to complications with vascular access.^31,36^ Stewardship of oral antibiotics could also be improved in this cohort by use of amoxicillin in place of co-amoxiclav (as an appropriate and palatable oral stepdown for GAS) for ongoing therapy.

VZV and influenza are vaccine-preventable illnesses known to predispose to iGAS infection,^37,38^ with influenza A/B detected in 8.1% of children and VZV preceding 2.7% of iGAS cases in this study. From September 2022 to January 2023, influenza vaccine uptake in England was only 56% in primary school-aged children and 27% in secondary school-aged children. The UK influenza vaccination program does not routinely include children under 2 years.^39^ A recommendation to add VZV vaccination to the UK schedule was given by the Joint Committee on Vaccination and Immunisation in November 2023,^40^ but administration is currently only advised by UKHSA when VZV is cocirculating with GAS infections in a nursery/preschool setting.^41^ VZV cases may be underestimated since data collection ceased before the usual seasonal VZV peak.

The proportion of viral coinfections recorded is likely to be an underestimate, as 17.3% of children did not have an extended respiratory pathogen multiplex PCR sent. While it is not possible to directly infer that cases occurred due to preceding viral infection, given what is known about VZV/influenza and iGAS, it is accepted that any preceding respiratory viral infection could interrupt mucosal integrity and increase the risk of secondary infection.^42^ It is important both to promote high coverage of existing vaccines as well as encourage progression on licensing new vaccines. For example, RSV maternal vaccine/infant monoclonal candidates have been recommended by the Joint Committee on Vaccination and Immunisation,^43^ and a hMPV vaccine candidate recently achieved good safety/tolerability profiles in phase one trials.^44^ With RSV detected in 17.3% and hMPV in 19.5% cases in this study, widespread and effective vaccination could potentially have an impact on GAS PPE burden, epidemiology should be monitored closely following any such intervention.

There are some imitations to our data. Data collection was via invitation rather than mandatory, potentially leading to a bias toward more severe cases from tertiary centers with Respiratory, Infection or PICU specialists. Given that our case fatality rate was markedly lower than recorded in UKHSA data, it appears we were able to detect a wide spectrum of disease severity, although patients dying at home with concurrent iGAS sepsis before chest x-ray may have been missed by this study. Data are observational rather than randomized, and while patients recovered after either chest drain or VATS procedure, it is worth noting that no children receiving VATS as their first intervention required a second intervention, and that fewer children receiving fibrinolytics alongside pleural drain required a second drain. Neither of these comparisons reached significance, therefore we cannot comment on the advantages/disadvantages of either VATS or pleural drain to assist with the equipoise on optimal surgical intervention.^45,46^ Although diagnoses can take time to be confirmed with molecular methods, early consideration of suspected case notification and appropriate post-exposure prophylaxis for close contacts of suspected cases is key.^41^ Strengths of this study include the collaboration of professional networks in rapid provision of valuable detail not previously available on demographics, presenting features and management of GAS pneumonia with PPE at a time of ongoing increased disease activity, and dissemination of this information to UKHSA and national bodies. It highlights the value of molecular testing both for GAS and for respiratory viruses, the need for clearer antibiotic guidance, improved antimicrobial stewardship and potential opportunities for disease prevention in the form of vaccination against viral preceding or coinfection.

### ACKNOWLEDGMENTS

*Members of the Group A Streptococcal Disease Consortium: Andrew Riordan, Alder Hey Children’s NHS Foundation Trust; Tembe Carveth Johnson, Barts Health NHS Trust; Juliette Oakley, Stefania Vergnano, Katharine Pike, Jolanta Bernatoniene, University Hospitals Bristol and Weston NHS Foundation Trust; David Inwald, Trust Donna McShane, Cambridge University Hospitals NHS Foundation Trust; Tan Ciang Sang, Guy’s and St Thomas’ NHS Foundation Trust; Thomas Bridge, Newcastle Hospitals NHS Foundation Trust; Stephanie Kuek, Sara Farah, Elise Randle, Rossa Brugha, Seilesh Kadambari, Garth Dixon, James Hatcher, Great Ormond Street Hospital for Children NHS Foundation Trust; Ismail Elghuwael, Kings College Hospital NHS Foundation Trust; Srini Bandi, Sharon Koo, University Hospitals of Leicester NHS Trust; Ayoade Adesina, Leeds Teaching Hospitals NHS Trust; Aditi Sinha, Lucy Hoskyns, Paddy McMaster, Manchester University NHS Foundation Trust; Anjay Pillai, Grace Kuruvilla, Norwich and Norfolk University Hospitals NHS Foundation Trust; Charlotte Kilpatrick, Trust Patrick Davies, Nottingham University Hospitals NHS Trust; Sheena Gordon, Katy Fidler, University Hospitals Sussex NHS Foundation Trust; Sarah Kavanagh, Rebecca Peto, Guy’s and St Thomas’ NHS Foundation Trust; Naomi Kirk, Lynne Speirs, James Finlay, Belfast Health and Social Care Trust; Katherine Longbottom, NHS Greater Glasgow and Clyde; Emily Hayes, Sheffield Children’s NHS Foundation Trust; Simon Drysdale, Ines Hormigo, Paula Devesa, St George’s University Hospitals NHS Foundation Trust; Giulia Lorenzetti, Asrar Bakar, Nicholas Alexander, Rebecca Mitting, Thomas Bycroft, Imperial College Healthcare NHS Trust; Michelle Rutter, Rachel Brampton, Naomi Haynes, Alison Garde, Lucy Everitt, Gabriella Watson, University Hospital Southampton NHS Foundation Trust; Andrew Ives, Jeremy Hull, Sarah-Jane Bowen, Oxford University Hospitals NHS Foundation Trust; Laura Walsh, Melissa Llano, Public Health Scotland; Paula Blomquist, Deepti Kumar, UK Health Security Agency.*

## Supplementary Material


