# Supplementary material for: Epidemiology and Management of Pediatric Group A Streptococcal Pneumonia With Parapneumonic Effusion: An Observational Study
Source: Pediatr Infect Dis J. 2024 Aug 9;43(9):841–50. doi: 10.1097/INF.0000000000004418 (PMC11319078; doi:10.1097/INF.0000000000004418)
Supplement: Supplementary file 1 [file inf-43-841-s001.pdf]

Supplemental Digital Content 1. Members of the Group A Streptococcal Disease Consortium

**First name:**	**Last name:**	**Affiliation:**
Andrew	Riordan	Alder Hey Children's NHS Foundation Trust
Tembe	Carveth-Johnson	Barts Health NHS Trust
Juliette	Oakley	University Hospitals Bristol and Weston NHS Foundation Trust
Stefania	Vergnano	University Hospitals Bristol and Weston NHS Foundation Trust
Katharine	Pike	University Hospitals Bristol and Weston NHS Foundation Trust
Jolanta	Bernatoniene	University Hospitals Bristol and Weston NHS Foundation Trust
David	Inwald	Cambridge University Hospitals NHS Foundation Trust
Donna	McShane	Cambridge University Hospitals NHS Foundation Trust
Tan	Ciang Sang	Guy's and St Thomas' NHS Foundation Trust
Thomas	Bridge	Newcastle Hospitals NHS Foundation Trust
Stephanie	Kuek	Great Ormond Street Hospital for Children NHS Foundation Trust
Sara	Farah	Great Ormond Street Hospital for Children NHS Foundation Trust
Elise	Randle	Great Ormond Street Hospital for Children NHS Foundation Trust
Rossa	Brugha	Great Ormond Street Hospital for Children NHS Foundation Trust
Seilesh	Kadambari	Great Ormond Street Hospital for Children NHS Foundation Trust
Garth	Dixon	Great Ormond Street Hospital for Children NHS Foundation Trust
James	Hatcher	Great Ormond Street Hospital for Children NHS Foundation Trust
Ismail	Elghuwael	Kings College Hospital NHS Foundation Trust
Srini	Bandi	University Hospitals of Leicester NHS Trust
Sharon	Koo	University Hospitals of Leicester NHS Trust
Ayoade	Adesina	Leeds Teaching Hospitals NHS Trust
Aditi	Sinha	Manchester University NHS Foundation Trust
Lucy	Hoskyns	Manchester University NHS Foundation Trust
Paddy	McMaster	Manchester University NHS Foundation Trust
Anjay	Pillai	Norwich and Norfolk University Hospitals NHS Foundation Trust
Grace	Kuruvilla	Norwich and Norfolk University Hospitals NHS Foundation Trust
Charlotte	Kilpatrick	Nottingham University Hospitals NHS Trust
Patrick	Davies	Nottingham University Hospitals NHS Trust
Sheena	Gordon	University Hospitals Sussex NHS Foundation Trust
Katy	Fidler	University Hospitals Sussex NHS Foundation Trust
Sarah	Kavanagh	Guy's and St Thomas' NHS Foundation Trust
Rebecca	Peto	Guy's and St Thomas' NHS Foundation Trust
Naomi	Kirk	Belfast Health and Social Care Trust
Lynne	Speirs	Belfast Health and Social Care Trust
James	Finlay	Belfast Health and Social Care Trust
Katherine	Longbottom	NHS Greater Glasgow and Clyde
Emily	Hayes	Sheffield Children's NHS Foundation Trust
Simon	Drysdale	St George's University Hospitals NHS Foundation Trust
Ines	Hormigo	St George's University Hospitals NHS Foundation Trust
Paula	Devesa	St George's University Hospitals NHS Foundation Trust
Giulia	Lorenzetti	Imperial College Healthcare NHS Trust
Asrar	Bakar	Imperial College Healthcare NHS Trust

Nicholas	Alexander	Imperial College Healthcare NHS Trust
Rebecca	Mitting	Imperial College Healthcare NHS Trust
Thomas	Bycroft	Imperial College Healthcare NHS Trust
Michelle	Rutter	University Hospital Southampton NHS Foundation Trust
Rachel	Brampton	University Hospital Southampton NHS Foundation Trust
Naomi	Haynes	University Hospital Southampton NHS Foundation Trust
Alison	Garde	University Hospital Southampton NHS Foundation Trust
Lucy	Everitt	University Hospital Southampton NHS Foundation Trust
Gabriella	Watson	University Hospital Southampton NHS Foundation Trust
Andrew	Ives	Oxford University Hospitals NHS Foundation Trust
Jeremy	Hull	Oxford University Hospitals NHS Foundation Trust
Sarah-Jane	Bowen	Oxford University Hospitals NHS Foundation Trust
Laura	Walsh	Public Health Scotland
Melissa	Llano	Public Health Scotland
Paula	Blomquist	UK Health Security Agency
Deepti	Kumar	UK Health Security Agency
