# Supplementary material for: Epidemiology and Management of Pediatric Group A Streptococcal Pneumonia With Parapneumonic Effusion: An Observational Study
Source: Pediatr Infect Dis J. 2024 Aug 9;43(9):841–50. doi: 10.1097/INF.0000000000004418 (PMC11319078; doi:10.1097/INF.0000000000004418)
Supplement: Supplementary file 2 [file inf-43-841-s002.pdf]

Supplemental Digital Content 2 - Methods for figures

Length of antibiotic course

Patients who had died were excluded from calculations for the length of antibiotic course. Only patients who had completed courses of intravenous (IV) antibiotics were included in the analysis for length of IV course; patients who remained inpatients on IV antibiotics, or for whom the length of course was unclear due to be transferred as an inpatient to another centre, were excluded. There was a single exception of one patient who had received intravenous antibiotics for 59 days, but continued intravenous antibiotics at the end date of the study. They were included in the analysis to capture the significant treatment burden associated with their invasive group A streptococcal disease. Except for this patient, only patients where the complete length of intravenous and oral antibiotic course was known were included in the calculation of total length of antibiotic course. Median and interquartile ranges were calculated and a violin plot created using the ggplot2[1] package in R[2] (R version 4.2.2) with the Zissou1 template from the Wes Anderson Palettes package.[3]

Antibiotics used

Any patient who had received antibiotics at any point, including those who had died, were included in this analysis. Python[4] (version 3.10) was used to disaggregate patient data by antibiotic. A heatmap was creating using the reshape[5] and ggplot2 packages in RStudio using the Zissou1 colour palette template.

Viral co-infections

Data on virus positivity from the 16 sentinel laboratories participating in the English Datamart Surveillance System was downloaded from the United Kingdom Health Security Agency (UKHSA) Official Statistics page.[6] Data on the number of tests performed from epidemiological week 40 in 2022 to week 15 in 2023 in the 0-4 and 5-14 year old age groups was provided by the Public Health Programme at UKHSA. Testing numbers were normalised

for all pathogens in the 0–4-year-old age group, and the proportion of tests in the 5–14-year-old age group relative to these scaled up or down accordingly. An assumption was made that in the 28 weeks that data was provided for there was equal number of tests per week, and this weekly count was multiplied by the reported positivity by virus by week to arrive at a pseudo-count in the 0-4 and 5-14 year age groups by week. A rolling fortnightly window was applied using the tidyverse[7] package in RStudio, and ridge plots creating using ggplot2.

All the raw data and code used are available in an anonymised, patient de-identified format on GitLab (https://git.ecdf.ed.ac.uk/twillia2/bpaiig_igas).

Results for Figures

Between epidemiologic week 40 of 2022 and week 15 of 2023 the 16 sentinel laboratories participating in the English Datamart Surveillance system submitted results for varying numbers of tests for the five pathogens included in Figure 2b (see Table, Supplemental Digital Content 3) Conall Watson, personal communication.

Making the assumption that these tests were evenly distributed across the 28 weeks of the season, a pseudo-count was created for each pathogen, by multiplying the number of weekly tests by percentage positive and dividing by 100, and summing the values for the 0-4 and 5-14 year age groups to create a total pseudo-count for all children <15 years old. For full details please refer to the GitLab page (https://git.ecdf.ed.ac.uk/twillia2/bpaiig_igas).

REFERENCES

1 Wickham H. ggplot2: Elegant Graphics for Data Analysis. Springer-Verlag New York, 2016 https://ggplot2.tidyverse.org.

2 RStudio. RStudio: integrated development for R. *RStudio, Inc, Boston, MA URL http//www rstudio com* 2015; **42**: 14.

3 Ram K. wesanderson. 2023. https://github.com/karthik/wesanderson.

4 Van Rossum G, Drake FL. Python 3 Reference Manual. Scotts Valley, CA: CreateSpace, 2009.

5 Wickham H. Reshaping data with the reshape package. *J Stat Softw* 2007; **21**. https://www.jstatsoft.org/v21/i12/.

6 UKHSA. National flu and COVID-19 surveillance data report: 4 May 2023 (week 18). 2023. https://assets.publishing.service.gov.uk/government/uploads/system/uploads/attachment_data/file/1154648/Weekly_Influenza_and_COVID19_report_data_w18_report.ods.

7 Wickham H, Averick M, Bryan J, *et al.* Welcome to the Tidyverse. *J open source Softw* 2019; **4**: 1686.

Supplemental Digital Content 3. Tests performed by pathogen in the 0-4 and 5-14 year age groups. Test numbers rounded to nearest 250.

**Pathogen**	**Number of tests in 0–4-year-olds**	**Number of tests in 5–14-year-olds**
Adenovirus	17,000	7,000
Influenza A	30,000	12,750
Human metapneumovirus	12,250	5,500
Rhinovirus	10,500	4,000
RSV	30,000	12,250

Supplemental Digital Content 4. Consort chart of surgical intervention by patient treated for group A streptococcal pneumonia with parapneumonic effusion

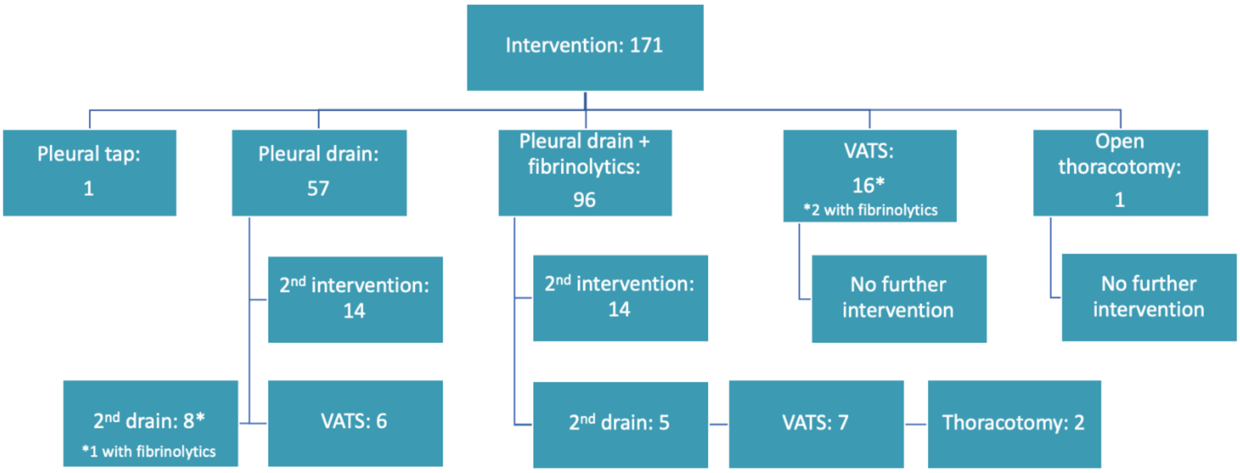

Key: VATS = video-assisted thoracoscopic surgery
